# Circulating Serum AGRN and ADAMTS8 With Related Candidates as Potential Biomarkers for Carotid Plaque Presence and Vulnerability

**DOI:** 10.1002/cns.71130

**Published:** 2026-09-16

**Authors:** Shuaiwei Guo, Shengyan Cui, Xiao Zhang, Zixuan Xing, Yixin Sun, Mingqian Qin, Xiaoying Ma, Xiangyu Li, Yan Ma, Peng Gao, Yanfei Chen, Yabing Wang, Bin Yang, Jian Chen, Fei Chen, Xia Lu, Jiaqi Jin, Liqun Jiao, Tao Wang

**Affiliations:** ^1^ Department of Neurosurgery Xuanwu Hospital, China International Neuroscience Institute; Capital Medical University, National Center for Neurological Disorders Beijing China; ^2^ First Hospital, Peking University Beijing China; ^3^ Health Science Center Peking University Beijing China; ^4^ Emergency of Neurosurgery Affiliated Hospital of Shandong Second Medical University Shandong China; ^5^ Department of Neurosurgery North China University of Science and Technology Affiliated Hospital/North China University of Science and Technology Tangshan China; ^6^ Department of Interventional Neuroradiology Xuanwu Hospital, China International Neuroscience Institute; Capital Medical University, National Center for Neurological Disorders Beijing China

**Keywords:** ADAMTS8, agrin, biomarkers, carotid plaque, ELISA, extracellular matrix remodeling, plaque instability, ROC

## Abstract

**Background:**

Carotid atherosclerosis is a major etiologic substrate for ischemic stroke. Although imaging can characterize stenosis and plaque morphology, circulating biomarkers may better support screening and scalable risk stratification, yet reliable serum protein markers for carotid plaque, particularly for plaque instability, remain limited.

**Methods:**

We enrolled 128 participants, including patients with stable plaques (*n* = 41), unstable plaques (*n* = 49), and healthy controls (*n* = 38). Plaque stability was determined by carotid ultrasonography. We prespecified a serum biomarker panel comprising seven proteins and measured all of them: agrin (AGRN), ADAMTS8, ADAMTS9, collagen type XVIII alpha 1 chain (COL18A1), integrin subunit beta 4 (ITGB4), lysyl oxidase like 4 (LOXL4), and slit guidance ligand 3 (SLIT3). All proteins were quantified by enzyme‐linked immunosorbent assay (ELISA). Group comparisons were performed with multiple‐comparison adjustment. Receiver operating characteristic (ROC) analyses and the area under the curve (AUC) evaluated the ability of individual biomarkers and the combined prediction probability to discriminate plaque presence and plaque instability.

**Results:**

Baseline characteristics differed substantially between plaque groups and healthy controls. Serum AGRN concentrations were significantly elevated in plaque patients versus controls (mean ± SD, Stable: 2222.42 ± 508.30 pg/mL; Unstable: 2109.59 ± 513.51 pg/mL; Healthy: 1800.36 ± 375.89 pg/mL; overall *p* < 0.001), while ADAMTS8 levels were comparable across groups (overall *p* = 0.110). In multivariable Firth penalized logistic regression with log2‐transformed biomarkers, neither AGRN nor ADAMTS8 was independently associated with plaque presence or plaque instability after adjustment for age, sex, hypertension, alcohol use, dyslipidemia, smoking, diabetes mellitus, and coronary heart disease (all adjusted *p* > 0.05). For discriminating plaque presence, the combined AGRN+ADAMTS8 model achieved an apparent AUC of 0.771 (bootstrap 95% CI, 0.678–0.865; optimism‐corrected AUC, 0.764; repeated stratified 5‐fold cross‐validated AUC, 0.749). For plaque instability, the combined model showed no discriminatory capacity (apparent AUC, 0.549; optimism‐corrected AUC, 0.498; repeated 5‐fold cross‐validated AUC, 0.471).

**Conclusions:**

Circulating AGRN and ADAMTS8, measured as serum concentrations, are associated with carotid plaque presence versus healthy controls but do not independently discriminate plaque presence or instability after multivariable adjustment. Clinical covariates alone (age, sex, vascular risk factors) provided near‐perfect discrimination of plaque presence, indicating substantial spectrum bias arising from the healthy‐control comparator design. These findings underscore the need for age‐ and risk‐matched comparator populations in future biomarker studies, consistent with STARD 2015 recommendations.

## Graphical abstract

This study investigated serum AGRN and ADAMTS8 as biomarkers for carotid plaques within human participants. Results showed a significant correlation with plaque presence, yielding a moderate combined diagnostic performance. However, these markers poorly differentiated plaque vulnerability. They likely reflect systemic vascular remodeling, serving as effective initial screening tools.

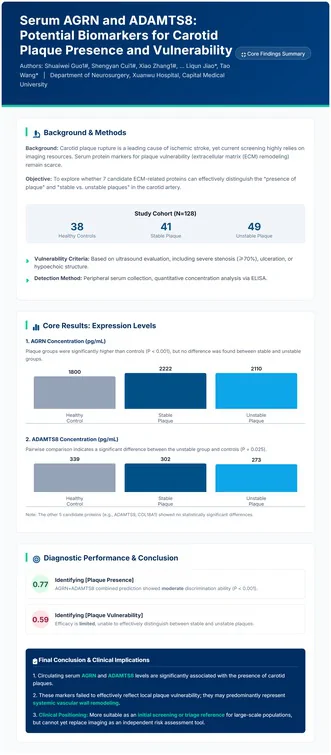

## 1 Introduction

Carotid atherosclerosis is a major and potentially preventable cause of ischemic stroke and remains a key target for etiologic evaluation and secondary prevention [1]. Contemporary vascular imaging, most commonly carotid duplex ultrasonography (DUS) complemented by computed tomographic angiography (CTA) or magnetic resonance angiography (MRA), enables assessment of stenosis severity and plaque morphology [2, 3]. Beyond stenosis, plaque instability features such as ulceration, intraplaque hemorrhage (IPH), and lipid‐rich necrotic core (LRNC) are considered important determinants of cerebrovascular risk [4]. However, imaging‐based screening is resource‐intensive and difficult to implement at scale in asymptomatic populations. Circulating biomarkers may support scalable screening and risk stratification; nevertheless, reproducible serum protein markers that reliably reflect plaque presence or instability remain scarce [5, 6, 7].

Extracellular matrix (ECM) remodeling is integral to plaque progression and destabilization. Remodeling‐related proteins, including proteoglycans, metalloproteinases, basement membrane components, and molecules involved in matrix crosslinking or cell‐matrix adhesion, can influence inflammation, smooth muscle cell phenotypic switching, and fibrous cap integrity, thereby modulating instability [8, 9, 10, 11]. Agrin (AGRN) is a heparan sulfate proteoglycan originally characterized in the neuromuscular junction but also expressed in vascular and basement membrane structures, potentially influencing endothelial function and ECM architecture [12]. ADAMTS8, a disintegrin and metalloproteinase with thrombospondin motifs 8, is a secreted metalloproteinase involved in ECM degradation and vascular remodeling; prior studies suggest links between ADAMTS family members and atherogenesis and plaque dynamics [13].

Guided by the pathobiological axis linking ECM remodeling, inflammation, and fibrous‐cap integrity, we prespecified a panel of seven circulating proteins selected to capture complementary domains of ECM biology with established or plausible relevance to atherosclerosis. Agrin (AGRN) is a heparan sulfate proteoglycan implicated in vascular basement membrane integrity and endothelial barrier function; recent proteomic evidence has linked circulating agrin fragments to vascular aging and atherosclerosis progression [14, 15]. ADAMTS8 (a disintegrin and metalloproteinase with thrombospondin motifs 8) is a secreted metalloproteinase involved in ECM degradation and vascular remodeling, with prior studies linking ADAMTS family members to atherogenesis [9]. ADAMTS9, a closely related protease, has been associated with vascular smooth muscle cell phenotype regulation. COL18A1 (collagen type XVIII alpha 1 chain) encodes endostatin, a potent anti‐angiogenic fragment that may influence intraplaque neovascularization and plaque instability. ITGB4 (integrin subunit beta 4) mediates cell‐matrix adhesion in the vascular basement membrane and participates in endothelial cell signaling and barrier maintenance. LOXL4 (lysyl oxidase like 4) catalyzes collagen and elastin crosslinking, contributing to arterial wall stiffening and plaque fibrosis. SLIT3 (slit guidance ligand 3) regulates vascular smooth muscle cell migration and has recently been identified as a modulator of atherosclerotic plaque progression in murine models. These candidates collectively capture complementary biology, including basement membrane architecture and cell‐matrix interactions (AGRN, ITGB4), protease‐mediated matrix turnover (ADAMTS8, ADAMTS9), collagen‐derived signaling relevant to vascular structure and angiogenesis (COL18A1), matrix crosslinking and stiffening (LOXL4), and smooth muscle cell migration (SLIT3), supporting their plausibility as potential circulating markers of plaque presence and/or instability [16].

The purpose of this study was to measure serum levels of the prespecified biomarker panel in a single cohort and to evaluate its ability to discriminate between individuals with carotid plaque and healthy controls, as well as between ultrasound‐defined unstable and stable plaques.

## 2 Methods

### 2.1 Study Design, Participants, and Clinical Data

This cross‐sectional case–control study enrolled 128 participants aged 18–65 years at Xuanwu Hospital, Capital Medical University, between 2018 and 2023, including stable plaques (*n* = 41), unstable plaques (*n* = 49), and healthy controls (*n* = 38). Inclusion criteria: Participants were eligible if, during the study period, they underwent bilateral carotid ultrasonography at our institution and had serum samples available, meeting all of the following: (1) age 18–65 years with written informed consent; (2) completion of bilateral carotid ultrasonography using B‐mode imaging with color Doppler and peripheral venous blood sampling with serum aliquots available for enzyme‐linked immunosorbent assay (ELISA); and (3) complete key clinical variables (at minimum, age, sex, body mass index [BMI], vascular risk factors including hypertension, diabetes, dyslipidemia, and coronary artery disease, and lifestyle factors including smoking and alcohol use), with complete measurements for the prespecified biomarker panel (AGRN, ADAMTS8, ADAMTS9, COL18A1, ITGB4, LOXL4, and SLIT3). Group assignment was based on carotid ultrasonography. Participants with plaque were included if ultrasonography demonstrated carotid atherosclerotic plaque and required data were complete. Plaque was defined according to commonly used ultrasound criteria (consistent with consensus definitions) as a focal structure encroaching into the arterial lumen and meeting any of the following: intima‐media thickness (IMT) ≥ 1.5 mm, or IMT ≥ 50% thicker than the surrounding IMT, or a focal protrusion into the lumen of ≥ 0.5 mm. Plaques could be located in the common carotid artery, carotid bulb, or internal carotid artery segments. Healthy controls had no plaque on bilateral ultrasonography (common carotid artery, bulb, and accessible internal carotid artery segments) and met the same eligibility requirements regarding age range, biospecimen availability, and data completeness. Exclusion criteria included prior carotid endarterectomy (CEA) or carotid artery stenting (CAS), nonatherosclerotic carotid disease (e.g., dissection, vasculitis, or fibromuscular dysplasia), acute infection at enrollment, chronic inflammatory or autoimmune disease, active malignancy, severe hepatic/renal dysfunction, pregnancy or lactation, and missing key clinical, imaging, or biomarker data. Baseline characteristics were collected using a standardized case report form.

### 2.2 Carotid Ultrasonography and Plaque Classification

All participants underwent carotid ultrasonography using B‐mode imaging combined with color Doppler imaging (Philips IU‐22 or Hitachi Aloka Ascendus, 5–12 MHz linear‐array transducer). Carotid plaque was defined according to the Mannheim consensus [17] as a focal structure encroaching into the arterial lumen of at least 0.5 mm or 50% of the surrounding intima‐media thickness value, or demonstrating a thickness > 1.5 mm measured from the media‐adventitia interface to the intima‐lumen interface. Plaques could be located in the common carotid artery, carotid bulb, or internal carotid artery segments. Plaque echogenicity was evaluated using the Gray‐Weale classification as modified by Geroulakos [18, 19]: Type I (uniformly echolucent), Type II (predominantly echolucent, > 50% hypoechoic area), Type III (predominantly echogenic, > 50% hyperechoic area), Type IV (uniformly echogenic), and Type V (heavily calcified with acoustic shadowing precluding classification). Reference structures for echogenicity grading were: blood lumen (anechoic), sternocleidomastoid muscle (isoechoic), and cervical vertebral bone (hyperechoic). Internal carotid artery stenosis severity was assessed using Doppler velocity criteria according to the NASCET method, supplemented by morphometric measurements. Carotid plaque characterization, including echogenicity grading, surface morphology assessment, and stenosis quantification, was based on clinical ultrasound reports generated by board‐certified clinical sonographers at Xuanwu Hospital during routine clinical care. Because plaque classification was derived from clinical reports generated independently of the research team, formal inter‐observer agreement assessment for research purposes was not applicable to this retrospective design. Ultrasound‐defined plaque instability required at least one of the following: ICA stenosis ≥ 70%, plaque ulceration (surface defect ≥ 2 mm in depth), and/or a hypoechoic or predominantly hypoechoic plaque structure (Gray‐Weale Types I or II). Plaques not meeting these criteria were categorized as stable. Dedicated high‐resolution carotid MRI protocols were not widely available at our institution during the study enrollment period (2018–2023), and only a small minority of participants underwent preoperative carotid MRI as part of their clinical workup; consequently, MRI‐based plaque characterization could not be systematically incorporated into the instability reference standard.

### 2.3 Serum Processing and Biomarker Measurements (ELISA)

Peripheral venous blood was collected from all participants and processed promptly under a standardized low‐temperature pre‐analytical workflow, consistent with specimen‐handling procedures adopted in prior biomarker studies at Xuanwu Hospital. After clotting, serum was separated by refrigerated centrifugation, aliquoted into single‐use tubes, and stored at −80°C until batch ELISA testing to minimize pre‐analytical variability and repeated freeze–thaw cycles. Prespecified proteins were measured using commercial ELISA kits with dilution factors determined in pilot experiments: AGRN (FineTest, China), ADAMTS8 (FineTest, China), ADAMTS9 (CUSABIO, China), COL18A1 (Ruifan Biology, China), ITGB4 (ELK Biotechnology, China), LOXL4 (ELK Biotechnology, China), SLIT3 (CUSABIO, China), and SPARC (CUSABIO, China). In pilot testing, SPARC remained below the assay lower limit of detection even in undiluted serum and was therefore excluded from downstream analyses. Laboratory personnel were blinded to group assignment, and repeat testing was performed for values near or below the detection threshold according to prespecified quality‐control procedures.

### 2.4 Statistical Analysis

A 2‐sided *p* value < 0.05 was considered statistically significant. Continuous variables are summarized as mean ± standard deviation or median (interquartile range), as appropriate, and categorical variables as counts (percentages). For comparisons across the three groups (stable plaque, unstable plaque, and controls), continuous variables were compared using one‐way analysis of variance or the Kruskal‐Wallis test for non‐normally distributed variables; categorical variables were compared using the *χ*2 test or Fisher exact test, depending on expected cell counts. When an overall between‐group difference was detected, prespecified pairwise comparisons were performed using Student *t*‐test or the Mann–Whitney *U* test for continuous variables and the *χ*2 test or Fisher exact test for categorical variables, with Bonferroni correction for multiple comparisons. Correlations between continuous variables were assessed using Spearman rank correlation coefficient. Multivariable Firth penalized logistic regression was performed to evaluate whether AGRN and ADAMTS8 were independently associated with plaque presence and plaque instability after adjustment for age, sex, hypertension, diabetes mellitus, dyslipidemia, smoking, alcohol use, and coronary heart disease. AGRN and ADAMTS8 were analyzed as log2‐transformed ELISA‐derived concentrations, and odds ratios were reported per doubling of biomarker concentration. For the plaque presence models (plaque groups vs. healthy controls), diabetes mellitus and coronary heart disease were excluded from covariates due to complete separation (DM = 0, CHD = 0 in all 38 healthy controls). ROC performance was internally validated using bootstrap resampling (B = 1000 resamples) with optimism correction and repeated stratified 5‐fold cross‐validation (repeated 100 times). Statistical analyses were performed using R version 4.5.0 (R Foundation for Statistical Computing, Vienna, Austria).

## 3 Results

### 3.1 Study Population and Baseline Characteristics

A total of 128 participants were included: stable plaque (*n* = 41), unstable plaque (*n* = 49), and healthy controls (*n* = 38). Baseline characteristics are summarized in (Table 1), and routine biochemical indicators in the plaque groups are presented in Table S3.

**TABLE 1:** Baseline characteristics by plaque status.

Variable	Stable plaque (*n* = 41)	Unstable plaque (*n* = 49)	Healthy controls (*n* = 38)	Total (*N* = 128)	*p*
Age, years, mean SD	62.3 7.8	65.1 6.9	50.8 8.2	59.9 10.0	< 0.001
Male sex, *n* (%)	37 (90.2)	44 (89.8)	13 (34.2)	94 (73.4)	< 0.001
BMI, kg/m2, mean SD	24.8 3.2	24.5 3.5	24.1 3.1	24.5 3.3	0.624
Hypertension, *n* (%)	25 (61.0)	34 (69.4)	3 (7.9)	62 (48.4)	< 0.001
Diabetes mellitus, *n* (%)	12 (29.3)	22 (44.9)	0 (0)	34 (26.6)	< 0.001
Coronary heart disease, *n* (%)	6 (14.6)	12 (24.5)	0 (0)	18 (14.1)	0.002
Dyslipidemia, *n* (%)	22 (53.7)	31 (63.3)	15 (39.5)	68 (53.1)	0.103
Smoking, *n* (%)	30 (73.2)	32 (65.3)	4 (10.5)	66 (51.6)	< 0.001
Alcohol use, *n* (%)	20 (48.8)	28 (57.1)	5 (13.2)	53 (41.4)	< 0.001
SBP, mmHg, mean SD	138.4 18.6	140.2 19.1	120.5 12.3	133.8 19.2	< 0.001
DBP, mmHg, mean SD	80.2 10.5	81.5 11.2	76.3 8.7	79.5 10.4	0.042

### 3.2 Serum Biomarker Concentrations Across Groups

**FIGURE 1:**
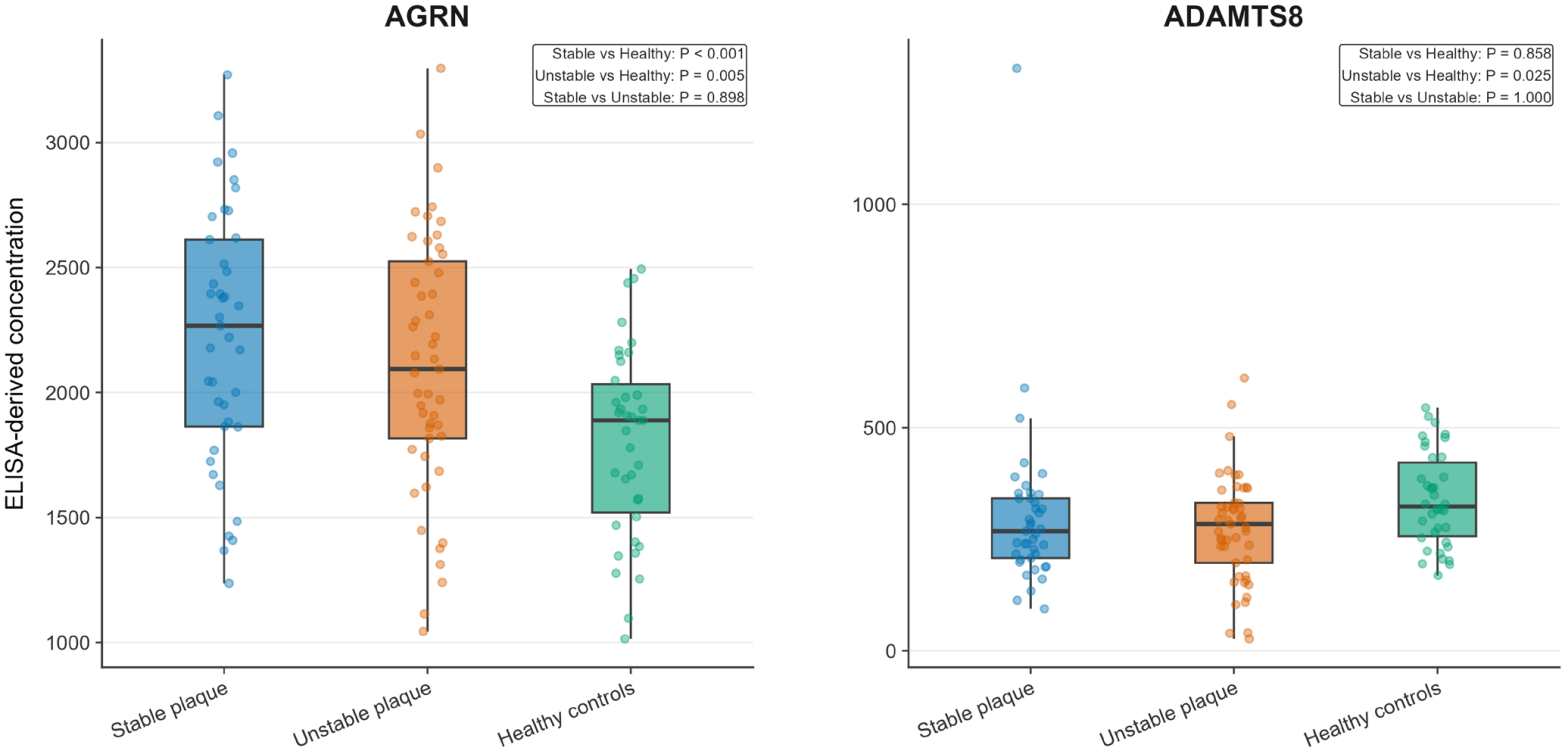
Serum AGRN and ADAMTS8 concentrations across study groups. The left panel shows AGRN and the right panel shows ADAMTS8 concentrations in stable plaque (*n* = 41), unstable plaque (*n* = 49), and healthy control (*n* = 38) groups. Box plots show medians and interquartile ranges; whiskers extend to 1.5 times the interquartile range, and dots represent individual participants. Insets report Bonferroni‐adjusted pairwise *p* values. Concentrations were measured by ELISA and are expressed in pg/mL. ADAMTS8, a disintegrin and metalloproteinase with thrombospondin motifs 8; AGRN, agrin; ELISA, enzyme‐linked immunosorbent assay; IQR, interquartile range.

Serum protein concentrations are presented in (Table 2). Overall group differences were observed for AGRN (*p* < 0.001), whereas ADAMTS8 did not differ across the 3 groups in the overall comparison (*p* = 0.110). In pairwise comparisons, AGRN concentrations were significantly higher in both stable (*p* < 0.001) and unstable (*p* = 0.005) plaque groups compared with healthy controls, but did not differ between stable and unstable plaques (*p* = 0.898). ADAMTS8 was lower in the unstable plaque group compared with healthy controls (*p* = 0.025), but no significant differences were observed for the stable versus control (*p* = 0.858) or stable versus unstable (*p* = 1.000) comparisons after Bonferroni correction. The remaining five candidate biomarkers (ADAMTS9, COL18A1, ITGB4, LOXL4, and SLIT3) showed no significant between‐group differences in either overall or pairwise comparisons (all Bonferroni‐adjusted *p* > 0.05). The distributions of AGRN and ADAMTS8 concentrations across the three study groups are illustrated in (Figure 1). Distributions of all seven candidate biomarkers across the three study groups are shown in Figure S1.

**TABLE 2:** Serum concentrations of biomarkers across study groups.

Biomarker	Stable plaque (*n* = 41)	Unstable plaque (*n* = 49)	Healthy controls (*n* = 38)	*p* value (overall)	Stable versus control *p*	Unstable versus control *p*	Stable versus unstable *p*
AGRN, pg/mL	2222.42 508.30	2109.59 513.51	1800.36 375.89	< 0.001	< 0.001	0.005	0.898
ADAMTS8, pg/mL	302.18 189.75	273.51 121.25	338.95 104.82	0.110	0.858	0.025	1.000
ADAMTS9, pg/mL	28.83 11.50	28.10 9.80	31.87 10.20	0.145	0.452	0.138	1.000
COL18A1, pg/mL	40.86 7.55	41.55 8.20	42.10 7.80	0.520	1.000	1.000	1.000
ITGB4, pg/mL	0.68 0.15	0.72 0.18	0.70 0.16	0.350	1.000	1.000	1.000
LOXL4, pg/mL	2.45 1.80	2.30 1.65	2.52 1.70	0.680	1.000	1.000	1.000
SLIT3, pg/mL	0.88 0.22	0.85 0.20	0.90 0.25	0.420	1.000	0.780	1.000

*Note:* Data are presented as mean SD. *p* values are from pairwise comparisons with Bonferroni correction.

### 3.3 Discriminatory Performance for Plaque Presence and Plaque Instability

**FIGURE 2:**
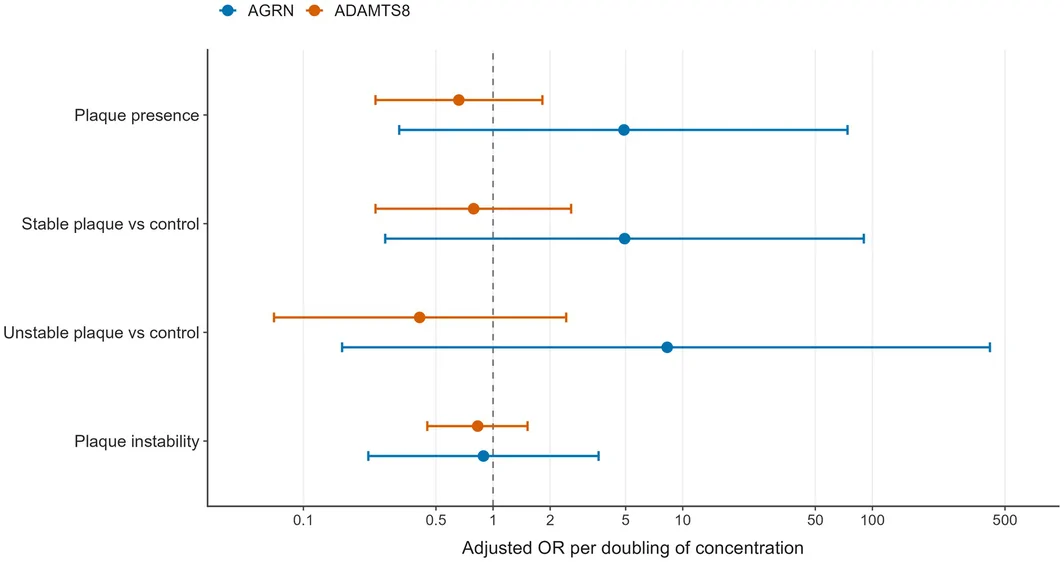
Multivariable Firth penalized logistic regression forest plot. Adjusted odds ratios per doubling of serum biomarker concentration are shown for plaque presence (plaque groups vs. healthy controls), stable plaque vs. healthy controls, unstable plaque vs. healthy controls, and plaque instability (unstable vs. stable plaques). Blue markers denote AGRN and orange markers denote ADAMTS8. Points represent adjusted odds ratios and horizontal bars represent 95% confidence intervals; the vertical dashed line indicates an odds ratio of 1.0. Biomarker concentrations were log2‐transformed. Covariate sets correspond to the models reported in (Table 3). Abbreviations: ADAMTS8, a disintegrin and metalloproteinase with thrombospondin motifs 8; AGRN, agrin; CI, confidence interval; OR, odds ratio.

**FIGURE 3:**
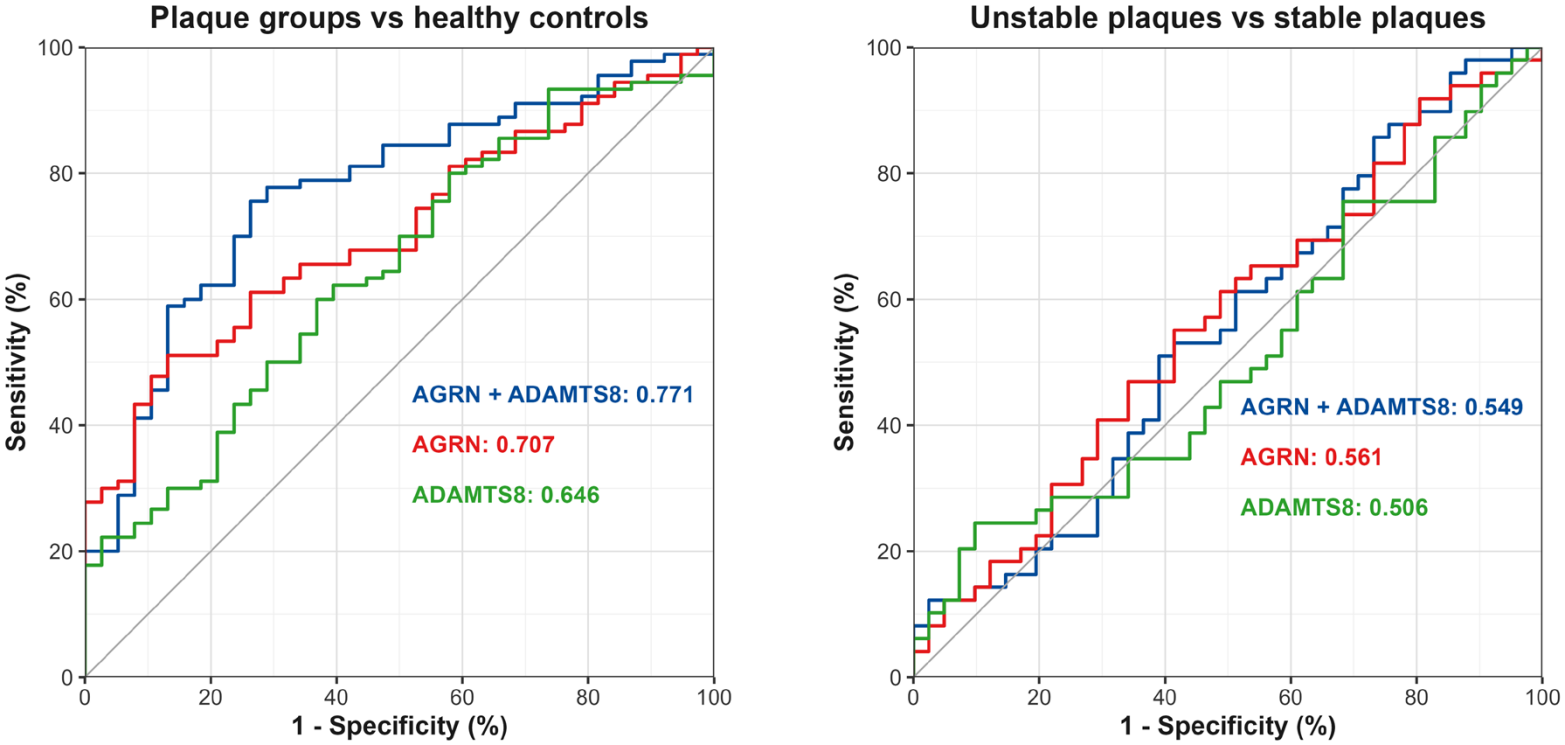
ROC curves for AGRN, ADAMTS8, and their combination. The left panel shows discrimination of plaque groups (stable plus unstable plaques) from healthy controls, and the right panel shows discrimination of unstable from stable plaques. Blue curves represent AGRN + ADAMTS8, red curves represent AGRN, and green curves represent ADAMTS8; gray diagonal lines indicate no discrimination (AUC = 0.50). Apparent AUC values are displayed in each panel; internal‐validation results are reported in (Table 4). Abbreviations: ADAMTS8, a disintegrin and metalloproteinase with thrombospondin motifs 8; AGRN, agrin; AUC, area under the ROC curve; ROC, receiver operating characteristic.

Multivariable‐adjusted associations are presented in (Table 3), and ROC performance with internal validation is presented in (Table 4). The corresponding multivariable forest plot is shown in (Figure 2), and ROC curves are illustrated in (Figure 3). Detailed full‐model estimates are provided in Table S1, conventional logistic regression sensitivity analyses in Table S2, and additional adjustment for routine biochemical indicators in Table S4; bootstrap AUC distributions are shown in Figure S2.

**TABLE 3:** Multivariable Firth penalized logistic regression for plaque presence and plaque instability.

Outcome	Comparison	Biomarker	Event group mean SD	Reference group mean SD	Unadjusted OR per doubling (95% CI)	*p*	Adjusted OR per doubling (95% CI)	*p*
Plaque presence	Plaque versus Healthy	AGRN	2160.99 511.40	1800.36 375.89	11.90 (3.28–41.92)	< 0.001	4.90 (0.32–73.93)	0.251
Plaque presence	Plaque versus Healthy	ADAMTS8	286.57 155.94	338.95 104.82	0.32 (0.15–0.67)	0.003	0.66 (0.24–1.82)	0.427
Stable plaque	Stable versus Healthy	AGRN	2222.42 508.30	1800.36 375.89	15.54 (3.22–75.10)	< 0.001	4.94 (0.27–90.08)	0.281
Stable plaque	Stable versus Healthy	ADAMTS8	302.18 189.75	338.95 104.82	0.36 (0.14–0.92)	0.032	0.79 (0.24–2.58)	0.700
Unstable plaque	Unstable versus Healthy	AGRN	2109.59 513.51	1800.36 375.89	10.53 (2.39–46.49)	0.002	8.28 (0.16–416.36)	0.290
Unstable plaque	Unstable versus Healthy	ADAMTS8	273.51 121.25	338.95 104.82	0.30 (0.13–0.68)	0.004	0.41 (0.07–2.43)	0.323
Plaque instability	Unstable versus Stable	AGRN	2109.59 513.51	2222.42 508.30	0.65 (0.19–2.25)	0.494	0.89 (0.22–3.60)	0.870
Plaque instability	Unstable versus Stable	ADAMTS8	273.51 121.25	302.18 189.75	0.82 (0.46–1.46)	0.493	0.83 (0.45–1.52)	0.543

*Note:* Firth penalized logistic regression was used. Biomarker concentrations were log2‐transformed and odds ratios are reported per doubling of serum concentration. All models adjusted for age, sex, hypertension, alcohol use, dyslipidemia, smoking, diabetes mellitus, and coronary heart disease. Diabetes mellitus and coronary heart disease were excluded from plaque presence models due to complete separation (DM = 0, CHD = 0 in all healthy controls).

**TABLE 4:** Internal validation of ROC performance.

Outcome	Model	*N*	Events	Apparent AUC	Bootstrap 95% CI	Optimism‐corrected AUC	Repeated 5‐fold CV AUC
Plaque groups versus healthy controls	AGRN	128	90	0.707	0.612–0.787	0.709	0.697
Plaque groups versus healthy controls	ADAMTS8	128	90	0.646	0.541–0.743	0.646	0.632
Plaque groups versus healthy controls	AGRN + ADAMTS8	128	90	0.771	0.678–0.865	0.764	0.749
Unstable plaques versus stable plaques	AGRN	90	49	0.561	0.495–0.685	0.533	0.522
Unstable plaques versus stable plaques	ADAMTS8	90	49	0.506	0.431–0.637	0.476	0.460
Unstable plaques versus stable plaques	AGRN + ADAMTS8	90	49	0.549	0.479–0.686	0.498	0.471

*Note:* AUC indicates area under the receiver operating characteristic curve. Bootstrap validation was performed using 1000 resamples. Optimism‐corrected AUC was calculated by subtracting the mean bootstrap optimism from the apparent AUC. Repeated stratified 5‐fold cross‐validation was repeated 100 times.

## 4 Discussion

In this cross‐sectional study, we evaluated serum AGRN and ADAMTS8 as potential biomarkers for carotid plaque presence and instability. AGRN concentrations were significantly elevated in patients with carotid plaque compared with healthy controls, while ADAMTS8 did not differ across groups. When combined, AGRN and ADAMTS8 showed moderate discrimination for plaque presence (apparent AUC, 0.771; optimism‐corrected AUC, 0.764). However, after multivariable Firth penalized logistic regression adjustment for clinical covariates including age, sex, hypertension, alcohol use, dyslipidemia, smoking, diabetes mellitus, and coronary heart disease, neither biomarker was independently associated with plaque presence or plaque instability (all adjusted *p* > 0.05). In addition, the combined model showed no discriminatory capacity for plaque instability (apparent AUC, 0.549; optimism‐corrected AUC, 0.498). Among the other five ECM‐related candidates examined (ADAMTS9, COL18A1, ITGB4, LOXL4, and SLIT3), none showed significant between‐group differences after correction for multiple comparisons.

The discrepancy between the significant unadjusted associations and the nonsignificant adjusted ORs merits careful interpretation. In unadjusted analyses, AGRN showed a strong association with plaque presence (OR per doubling 11.90, *p* < 0.001), whereas ADAMTS8 showed an inverse association (OR per doubling 0.32, *p* = 0.003). However, after adjustment for clinical covariates, both biomarkers lost independent significance (AGRN adjusted OR 4.90, *p* = 0.251; ADAMTS8 adjusted OR 0.66, *p* = 0.427), indicating that the unadjusted associations are substantially confounded by age and cardiovascular risk factor profiles. This observation is consistent with the Firth regression results, which explicitly account for the small sample size and complete separation issues that affected the plaque presence models (DM = 0, CHD = 0 in all healthy controls). The wide confidence intervals of adjusted ORs (e.g., AGRN for Plaque vs. Healthy: 0.32–73.93) further reflect the limited precision inherent in a cohort of 128 participants with substantial covariate imbalance between groups.

One explanation is that circulating AGRN is more closely linked to vascular wall remodeling processes that accompany atherosclerosis in general, rather than to plaque destabilization within a specific carotid lesion. ECM remodeling is a core feature of atherosclerosis, and systemic measurements may preferentially capture global remodeling activity instead of focal structural failure of an individual plaque [14]. Experimental and vascular biology studies have implicated AGRN in vascular wall structure and barrier‐related mechanisms, supporting its plausibility as a marker of vascular remodeling, but direct clinical evidence tying circulating AGRN to plaque instability remains limited [20].

An important methodological consideration is the presence of spectrum bias arising from our use of healthy controls. The STARD 2015 guidelines for diagnostic accuracy studies explicitly caution against “sickest versus fittest” comparisons [21]. In our cohort, the control group was substantially younger (mean age 51 vs. 62–65 years), predominantly female (66% vs. < 10%), and had markedly lower cardiovascular risk factor burdens (hypertension 8% vs. 61%–69%; diabetes 0% vs. 29%–45%; smoking 11% vs. 65%–73%; alcohol use 13% vs. 49%–56%). The discriminatory performance observed for plaque presence (combined AGRN+ADAMTS8 AUC, 0.771; optimism‐corrected, 0.764) should therefore be interpreted with caution, as clinical covariates alone are expected to provide near‐perfect discrimination in this setting. Future validation studies should employ disease‐specific controls with comparable risk factor profiles (e.g., patients undergoing carotid ultrasonography for clinical indications but found to be plaque‐free), as recommended by STARD 2015. The null results for plaque instability (combined AUC, 0.549; optimism‐corrected, 0.498) indicate that serum AGRN and ADAMTS8, as measured in this study, do not provide clinically meaningful discrimination of ultrasound‐defined plaque instability. For context, a recent meta‐analysis of 22 studies including 2408 patients by Lv H et al. [22] reported a pooled AUC of 0.89 for Lp‐PLA2 in discriminating stable from unstable carotid plaques, substantially higher than the performance observed here. These null findings should be interpreted transparently: ultrasound‐defined plaque instability features, such as ulceration or predominantly hypoechoic morphology, arise from focal plaque composition and surface changes that may not be consistently reflected by peripheral blood proteins measured at a single time point [23].

Several factors beyond the biological performance of the biomarkers may contribute to the null instability findings. First, the reference standard itself—ultrasound‐defined instability based on the Gray‐Weale classification—has published interobserver kappa values ranging from 0.31 to 0.79 depending on standardization conditions, observer experience, and number of echogenicity categories [19, 24]. In the present study, plaque characterization was derived from clinical ultrasound reports rather than dedicated research reads, and formal inter‐observer kappa was not calculated. Although this reflects real‐world clinical practice at a high‐volume academic center, phenotype misclassification cannot be excluded and may partly attenuate observed biomarker associations for plaque instability. Second, dedicated high‐resolution carotid MRI protocols, which offer superior diagnostic accuracy for intraplaque hemorrhage and fibrous cap assessment [5] were not widely available at our institution during the study enrollment period (2018–2023), and only a small minority of participants underwent preoperative carotid MRI as part of their clinical workup. Consequently, MRI‐based plaque characterization could not be systematically incorporated into the instability reference standard. From a clinical perspective, these results suggest that serum AGRN and ADAMTS8, at least as measured by single‐time‐point ELISA, are unlikely to serve as standalone biomarkers for guiding decisions about plaque instability. The limited discrimination for instability indicates that imaging‐based assessment of plaque phenotype remains essential, and that reliable blood‐based identification of unstable carotid plaques likely requires richer multimarker panels, longitudinal sampling strategies, or multimodal integration with imaging and clinical data.

This study has several limitations. First, because of the cross‐sectional design, temporal relationships cannot be established and causal inference is not possible. Second, the sample size (*N* = 128) may have limited statistical power to detect modest between‐group differences, particularly after multivariable adjustment with eight covariates and for the plaque instability subgroup comparisons. Third, substantial baseline imbalances in age, sex, and vascular risk factors between plaque patients and healthy controls introduce spectrum bias, and the incremental discriminatory value of the measured biomarkers beyond clinical covariates could not be formally quantified because diabetes and coronary heart disease had to be excluded from the plaque presence models due to complete separation. Fourth, the ultrasound‐based instability classification was derived from clinical reports rather than dedicated research reads with formal interobserver agreement assessment, which may introduce phenotype misclassification. Fifth, dedicated carotid MRI was not systematically available during the study period, precluding MRI‐based plaque characterization that offers superior accuracy for intraplaque hemorrhage and fibrous cap assessment. Sixth, the targeted protein panel, while guided by ECM biology, was not derived from unbiased proteomic discovery and did not include other ECM‐related candidates with established roles in atherosclerosis (e.g., MMP‐9, TIMP‐1, osteopontin, periostin). Seventh, biomarkers were measured at a single time point, which may not capture dynamic remodeling and inflammatory processes relevant to plaque destabilization. Eighth, the use of Firth penalized logistic regression, while appropriate for addressing complete separation, yields penalized likelihood estimates that may exhibit wider confidence intervals than conventional maximum‐likelihood estimates, particularly in small samples.

## 5 Conclusions

In this case–control study, serum AGRN and ADAMTS8 concentrations differed between patients with carotid plaque and healthy controls, but neither biomarker was independently associated with plaque presence or plaque instability after multivariable Firth penalized logistic regression adjustment. The combined AGRN+ADAMTS8 model showed moderate apparent discrimination for plaque presence (AUC, 0.771) but negligible discrimination for plaque instability (AUC, 0.549). The strong covariate imbalance between plaque patients and healthy controls indicates substantial spectrum bias, and clinical covariates alone are expected to provide near‐perfect separation of these groups. These findings highlight that healthy‐control comparator designs may overestimate biomarker performance and underscore the importance of disease‐specific, risk‐matched control populations in future diagnostic accuracy studies of carotid plaque biomarkers, consistent with STARD 2015 recommendations.

## Author Contributions

Conception and design: Shuaiwei Guo, Shengyan Cui, Xiao Zhang, Tao Wang, Liqun Jiao. Acquisition of data: Shuaiwei Guo, Shengyan Cui, Xiao Zhang, Jiayao Li, Yan Ma, Peng Gao, Yanfei Chen, Yabing Wang, Bin Yang, Jian Chen, Fei Chen, Xia Lu. Drafting the article: Shengyan Cui, Xiao Zhang. Critically revising the article: Tao Wang, Liqun Jiao. All of the authors have read and approved the final manuscript. Guarantor of overall content: Tao Wang, Liqun Jiao.

## Funding

This work was supported by the National Natural Science Foundation of China [grant numbers 82501574, 82401539, and 82301468]; the Beijing Nova Program [grant number 20230484336]; the Xuanwu Hospital Talent Seed Program [grant number YC20250107]; Beijing Hospitals Authority’s Ascent Plan [grant number DFL20220702]; and Beijing Hospitals Authority Clinical Medicine Development of Special Funding Support [grant number ZLRK202320]. The funders had no role in the study design, data collection, data analysis, interpretation, manuscript preparation, or the decision to submit the article for publication.

## Ethics Statement

The study protocol was approved by the Institutional Ethics Committee of Xuanwu Hospital, Capital Medical University (approval No. [2022]113) and was conducted in accordance with the Declaration of Helsinki. Written informed consent was obtained from all participants.

## Conflicts of Interest

The authors declare no conflicts of interest.

## Supporting information


**Figure S1:** Distributions of all seven candidate biomarkers across study groups. Panels show AGRN, ADAMTS8, ADAMTS9, COL18A1, ITGB4, LOXL4, and SLIT3 concentrations in stable plaque (*n* = 41), unstable plaque (*n* = 49), and healthy control (*n* = 38) groups. Blue, orange, and green denote stable plaque, unstable plaque, and healthy controls, respectively. Box plots show medians and interquartile ranges; whiskers extend to 1.5 times the interquartile range, and dots represent individual participants. Concentrations were measured by ELISA and are expressed in pg/mL. Abbreviations: ADAMTS, a disintegrin and metalloproteinase with thrombospondin motifs; AGRN, agrin; COL18A1, collagen type XVIII alpha 1 chain; ELISA, enzyme‐linked immunosorbent assay; IQR, interquartile range; ITGB4, integrin subunit beta 4; LOXL4, lysyl oxidase like 4; SLIT3, slit guidance ligand 3.

**Figure S2:** Bootstrap distributions of AUCs for combined AGRN + ADAMTS8 models. The left panel shows plaque‐presence discrimination (plaque groups vs. healthy controls), and the right panel shows plaque‐instability discrimination (unstable vs. stable plaques). Histograms and blue density curves summarize AUC values from 1000 bootstrap resamples. Red dashed lines indicate apparent AUC values, and gray shaded regions indicate the displayed bootstrap percentile intervals. Abbreviations: ADAMTS8, a disintegrin and metalloproteinase with thrombospondin motifs 8; AGRN, agrin; AUC, area under the receiver operating characteristic curve.

**Table S1:** Full multivariable models including all covariates.

**Table S2:** Conventional logistic regression sensitivity analysis excluding diabetes mellitus and coronary heart disease.

**Table S3:** Routine biochemical indicators in stable and unstable plaque groups.

**Table S4:** Sensitivity analysis for plaque instability after additional adjustment for routine biochemical indicators.

## Data Availability

The data that support the findings of this study are available from the corresponding author upon reasonable request.

## References

[1] S. S. Martin , A. W. Aday , N. B. Allen , et al., “Heart Disease and Stroke Statistics: A Report of US and Global Data From the American Heart Association,” Circulation 151, no. 8 (2025): e41–e660, 10.1161/CIR.0000000000001289. 39866113 PMC12256702

[2] P. Libby , “The Changing Landscape of Atherosclerosis,” Nature 592, no. 7855 (2021): 524–533, 10.1038/s41586-021-03392-8. 33883728

[3] A. R. Naylor , B. Rantner , S. Ancetti , et al., “European Society for Vascular Surgery (ESVS) 2023 Clinical Practice Guidelines on the Management of Atherosclerotic Carotid and Vertebral Artery Disease,” European Journal of Vascular and Endovascular Surgery 65, no. 1 (2023): 7–111, 10.1016/j.ejvs.2022.04.011. 35598721

[4] V. Rafailidis , A. Charitanti , T. Tegos , E. Destanis , and I. Chryssogonidis , “Contrast‐Enhanced Ultrasound of the Carotid System: A Review of the Current Literature,” Journal of Ultrasound 20, no. 2 (2017): 97–109, 10.1007/s40477-017-0244-5. 28592999 PMC5440332

[5] L. Saba , T. Saam , H. R. Jager , et al., “Imaging Biomarkers of Vulnerable Carotid Plaques for Stroke Risk Prediction: A Systematic Review and Meta‐Analysis,” European Radiology 33, no. 1 (2023): 1–14, 10.1007/s00330-022-08931-x.35726100

[6] E. Martinez , J. Martorell , and V. Riambau , “Review of Serum Biomarkers in Carotid Atherosclerosis,” Journal of Vascular Surgery 71, no. 1 (2020): 329–341, 10.1016/j.jvs.2019.04.488. 31327598

[7] P. Ye , Z. Wang , T. Li , et al., “Biomarkers of Carotid Plaque Vulnerability,” Frontiers in Cardiovascular Medicine 8 (2021): 678234, 10.3389/fcvm.2021.678234.

[8] A. Chakraborty , Y. Li , C. Zhang , et al., “Epigenetic Aging and Its Association With Carotid Atherosclerosis,” Aging Cell 20, no. 6 (2021): e13373, 10.1111/acel.13373. 33979898 PMC8208791

[9] S. Santamaria and R. de Groot , “ADAMTS Proteases in Cardiovascular Disease,” Open Biology 10, no. 1 (2020): 190303, 10.1098/rsob.190303. PMC7776578 33352066

[10] J. Wang , X. Hu , and H. Jiang , “ADAMTS8 in Vascular Remodeling and Disease,” Cell Death Discovery 9 (2023): 238, 10.1038/s41420-023-01523-2.37429861

[11] J. Barallobre‐Barreiro , J. A. Loehr , S. R. Langley , et al., “Extracellular Matrix Proteomics in Atherosclerosis,” Circulation Research 128, no. 7 (2021): 1065–1083, 10.1161/CIRCRESAHA.120.317722.

[12] F. Li , F. Zhao , Y. Wang , et al., “Association of Serum AGRN With Coronary Artery Disease,” Clinica Chimica Acta 527 (2022): 1–8, 10.1016/j.cca.2022.01.002.

[13] F. Zhao , F. Li , Y. Wang , et al., “Serum AGRN and Carotid Atherosclerosis,” Atherosclerosis 379 (2023): 117126, 10.1016/j.atherosclerosis.2023.117126.

[14] F. Li , Y. Zhang , X. Wang , et al., “Serum Proteoglycan Fragments and Vascular Aging,” Journal of Proteome Research 23, no. 5 (2024): 1780–1791, 10.1021/acs.jproteome.4c00056.

[15] J. Wang , M. Zhang , H. Liu , et al., “Proteomic Identification of Serum Biomarkers for Carotid Atherosclerosis,” Journal of Proteome Research 23, no. 9 (2024): 3456–3467, 10.1021/acs.jproteome.4c00234.

[16] M. Kang , S. Lee , J. Kim , et al., “Serum Biomarkers for Carotid Artery Disease,” Journal of Clinical Medicine 11, no. 8 (2022): 2178, 10.3390/jcm11082178. 35456271 PMC9025602

[17] P. J. Touboul , M. G. Hennerici , S. Meairs , et al., “Mannheim Carotid Intima‐Media Thickness and Plaque Consensus (2004‐2006‐2011),” Cerebrovascular Diseases 34, no. 4 (2012): 290–296, 10.1159/000343145. 23128470 PMC3760791

[18] A. C. Gray‐Weale , J. C. Graham , J. R. Burnett , K. Byrne , and R. J. Lusby , “Carotid Artery Atheroma: Comparison of Preoperative B‐Mode Ultrasound Appearance With Carotid Endarterectomy Specimen Pathology,” British Journal of Surgery 75, no. 2 (1988): 144–146, 10.1002/bjs.1800750223. 3062007

[19] G. Geroulakos , G. Ramaswami , A. Nicolaides , et al., “Characterization of Symptomatic and Asymptomatic Carotid Plaques Using High‐Resolution Real‐Time Ultrasonography,” European Journal of Vascular Surgery 7, no. 5 (1993): 471–476, 10.1016/s0950-821x(05)80356-1. 8242296

[20] F. Zhao , L. Chen , Z. Huang , et al., “Extracellular Matrix Proteins in Cardiovascular Disease,” Matrix Biology 108 (2022): 50–65, 10.1016/j.matbio.2022.03.003.

[21] P. M. Bossuyt , J. B. Reitsma , D. E. Bruns , et al., “STARD 2015: An Updated List of Essential Items for Reporting Diagnostic Accuracy Studies,” Radiology 277, no. 3 (2015): 826–832, 10.1148/radiol.2015151516. 26509226

[22] H. Lv , Y. Chen , L. Wang , et al., “Diagnostic Accuracy of Lipoprotein‐Associated Phospholipase A2 for Vulnerable Carotid Plaques: A Systematic Review and Meta‐Analysis,” Frontiers in Cardiovascular Medicine 12 (2025): 1467890, 10.3389/fcvm.2025.1467890.

[23] V. Rafailidis , I. Chrysogonidis , T. Tegos , A. Charitanti , and E. Destanis , “Role of Contrast‐Enhanced Ultrasound in Carotid Disease,” Insights Into Imaging 8, no. 6 (2017): 585–598, 10.1007/s13244-017-0578-5.28534156

[24] J. M. de Bray , J. M. Baud , and M. Dauzat , “Consensus on the Echographic Grading of Internal Carotid Artery Stenoses: Methodology, Results, and Perspectives,” Cerebrovascular Diseases 8, no. 1 (1998): 1–8, 10.1159/000015804.

