# Supplementary material for: Circulating Serum AGRN and ADAMTS8 With Related Candidates as Potential Biomarkers for Carotid Plaque Presence and Vulnerability
Source: CNS Neurosci Ther. 2026 Sep 16;32(9):e71130. doi: 10.1002/cns.71130 (PMC13580344; doi:10.1002/cns.71130)
Supplement: Supplementary file 1 [file CNS-32-e71130-s002.docx]

**Supplementary Table 1. Full multivariable models including all covariates**

| **Outcome** | **Comparison** | **Biomarker** | **Event group mean SD** | **Reference group mean SD** | **Adjusted OR per doubling** | **95% CI** | **P value** |
| --- | --- | --- | --- | --- | --- | --- | --- |
| **Plaque presence** | **Plaque vs healthy** | **AGRN** | **2160.99 511.40** | **1800.36 375.89** | **4.84** | **0.37-63.74** | **0.231** |
| **Plaque presence** | **Plaque vs healthy** | **ADAMTS8** | **286.57 155.94** | **338.95 104.82** | **0.67** | **0.25-1.78** | **0.421** |
| **Stable plaque** | **Stable vs healthy** | **AGRN** | **2222.42 508.30** | **1800.36 375.89** | **5.65** | **0.33-96.85** | **0.232** |
| **Stable plaque** | **Stable vs healthy** | **ADAMTS8** | **302.18 189.75** | **338.95 104.82** | **0.79** | **0.25-2.51** | **0.689** |
| **Unstable plaque** | **Unstable vs healthy** | **AGRN** | **2109.59 513.51** | **1800.36 375.89** | **6.40** | **0.18-233.51** | **0.312** |
| **Unstable plaque** | **Unstable vs healthy** | **ADAMTS8** | **273.51 121.25** | **338.95 104.82** | **0.44** | **0.10-2.02** | **0.291** |
| **Plaque instability** | **Unstable vs stable** | **AGRN** | **2109.59 513.51** | **2222.42 508.30** | **0.89** | **0.22-3.59** | **0.866** |
| **Plaque instability** | **Unstable vs stable** | **ADAMTS8** | **273.51 121.25** | **302.18 189.75** | **0.83** | **0.45-1.52** | **0.543** |

All models adjusted for age, sex, hypertension, alcohol use, dyslipidemia, smoking, diabetes mellitus, and coronary heart disease. Firth penalized logistic regression was used. Biomarker concentrations were log2-transformed and odds ratios are reported per doubling of serum concentration.
