# Supplementary material for: Circulating Serum AGRN and ADAMTS8 With Related Candidates as Potential Biomarkers for Carotid Plaque Presence and Vulnerability
Source: CNS Neurosci Ther. 2026 Sep 16;32(9):e71130. doi: 10.1002/cns.71130 (PMC13580344; doi:10.1002/cns.71130)
Supplement: Supplementary file 2 [file CNS-32-e71130-s003.docx]

**Supplementary Table 2. Conventional logistic regression sensitivity analysis excluding diabetes mellitus and coronary heart disease**

| **Outcome** | **Comparison** | **Biomarker** | **Adjusted OR per doubling** | **95% CI** | **P value** |
| --- | --- | --- | --- | --- | --- |
| **Plaque presence** | **Plaque vs Healthy** | **AGRN** | **6.38** | **0.31-132.26** | **0.231** |
| **Plaque presence** | **Plaque vs Healthy** | **ADAMTS8** | **0.52** | **0.17-1.66** | **0.271** |
| **Stable plaque** | **Stable vs Healthy** | **AGRN** | **5.19** | **0.12-228.65** | **0.394** |
| **Stable plaque** | **Stable vs Healthy** | **ADAMTS8** | **0.67** | **0.18-2.56** | **0.561** |
| **Unstable plaque** | **Unstable vs Healthy** | **AGRN** | **14.65** | **0.24-883.36** | **0.199** |
| **Unstable plaque** | **Unstable vs Healthy** | **ADAMTS8** | **0.17** | **0.02-1.56** | **0.119** |
| **Plaque instability** | **Unstable vs Stable** | **AGRN** | **0.79** | **0.20-3.21** | **0.745** |
| **Plaque instability** | **Unstable vs Stable** | **ADAMTS8** | **0.78** | **0.42-1.45** | **0.437** |

Sensitivity analyses using conventional (non-penalized) multivariable logistic regression with log2-transformed biomarker concentrations. Diabetes mellitus and coronary heart disease were excluded from all models to avoid complete separation. All models adjusted for age, sex, hypertension, alcohol use, dyslipidemia, and smoking.
