# Supplementary material for: Circulating Serum AGRN and ADAMTS8 With Related Candidates as Potential Biomarkers for Carotid Plaque Presence and Vulnerability
Source: CNS Neurosci Ther. 2026 Sep 16;32(9):e71130. doi: 10.1002/cns.71130 (PMC13580344; doi:10.1002/cns.71130)
Supplement: Supplementary file 3 [file CNS-32-e71130-s004.docx]

**Supplementary Table 4. Sensitivity analysis for plaque instability after additional adjustment for routine biochemical indicators**

| **Model** | **N** | **Events** | **Predictor** | **Adjusted OR (95% CI)** | **P value** |
| --- | --- | --- | --- | --- | --- |
| **Primary clinical model** | **90** | **49** | **AGRN, per doubling** | **0.89 (0.22-3.60)** | **0.870** |
| **Primary clinical model** | **90** | **49** | **ADAMTS8, per doubling** | **0.83 (0.45-1.52)** | **0.543** |
| **Sensitivity 1: clinical + CRP/LDL-C/glucose** | **90** | **49** | **AGRN, per doubling** | **0.76 (0.17-3.39)** | **0.722** |
| **Sensitivity 1: clinical + CRP/LDL-C/glucose** | **90** | **49** | **ADAMTS8, per doubling** | **0.74 (0.38-1.43)** | **0.367** |
| **Sensitivity 2: clinical + expanded biochemistry** | **90** | **49** | **AGRN, per doubling** | **0.76 (0.16-3.68)** | **0.735** |
| **Sensitivity 2: clinical + expanded biochemistry** | **90** | **49** | **ADAMTS8, per doubling** | **0.76 (0.39-1.46)** | **0.405** |

The outcome was plaque instability among plaque patients (unstable plaque = 1; stable plaque = 0). Firth penalized logistic regression was used. AGRN and ADAMTS8 were log2-transformed and odds ratios are reported per doubling of serum concentration. CRP was also log2-transformed. Model 1 (Primary clinical model) adjusted for age, sex, hypertension, diabetes mellitus, dyslipidemia, and smoking. Model 2 (Sensitivity 1) additionally adjusted for CRP, LDL-C, and blood glucose. Model 3 (Sensitivity 2) additionally adjusted for CRP, LDL-C, blood glucose, ALT, and creatinine.
