# Supplementary material for: Circulating Serum AGRN and ADAMTS8 With Related Candidates as Potential Biomarkers for Carotid Plaque Presence and Vulnerability
Source: CNS Neurosci Ther. 2026 Sep 16;32(9):e71130. doi: 10.1002/cns.71130 (PMC13580344; doi:10.1002/cns.71130)
Supplement: Supplementary file 4 [file CNS-32-e71130-s006.docx]

**Supplementary Table 3. Routine biochemical indicators in stable and unstable plaque groups**

| **Variable** | **Unit** | **Stable n** | **Stable mean SD** | **Stable median (IQR)** | **Unstable n** | **Unstable mean SD** | **Unstable median (IQR)** | **P value** |
| --- | --- | --- | --- | --- | --- | --- | --- | --- |
| **CRP** | **mg/L** | **41** | **4.81 9.80** | **0.97 (0.46-3.43)** | **49** | **2.31 3.29** | **0.92 (0.45-3.09)** | **0.635** |
| **Total cholesterol** | **mmol/L** | **41** | **3.41 1.13** | **3.35 (2.70-3.95)** | **49** | **3.66 0.97** | **3.59 (3.09-4.19)** | **0.115** |
| **Triglycerides** | **mmol/L** | **41** | **1.54 0.84** | **1.30 (1.05-1.79)** | **49** | **1.59 0.86** | **1.40 (1.02-1.91)** | **0.743** |
| **HDL-C** | **mmol/L** | **41** | **1.02 0.32** | **1.00 (0.84-1.18)** | **49** | **1.07 0.28** | **1.05 (0.85-1.23)** | **0.514** |
| **LDL-C** | **mmol/L** | **41** | **1.71 0.55** | **1.66 (1.27-2.13)** | **49** | **1.97 0.73** | **1.77 (1.51-2.31)** | **0.072** |
| **Blood glucose** | **mmol/L** | **41** | **6.27 2.94** | **5.42 (4.81-7.03)** | **49** | **6.56 2.07** | **5.87 (5.22-7.56)** | **0.208** |
| **ALT** | **U/L** | **41** | **26.63 32.45** | **18.00 (15.00-26.00)** | **49** | **29.53 16.29** | **27.00 (18.00-39.00)** | **0.032** |
| **AST** | **U/L** | **41** | **22.44 13.31** | **20.00 (17.00-24.00)** | **49** | **23.27 7.69** | **21.00 (17.00-29.00)** | **0.278** |
| **Creatinine** | **mol/L** | **41** | **69.37 11.46** | **70.00 (60.00-76.00)** | **49** | **76.06 29.79** | **69.00 (60.00-81.00)** | **0.749** |
| **Urea** | **mmol/L** | **41** | **5.68 1.57** | **5.44 (4.67-6.89)** | **49** | **5.83 1.48** | **5.53 (4.66-6.67)** | **0.662** |

Data are presented as mean SD and median (interquartile range). Between-group comparisons were performed using the Mann-Whitney U test. Biochemical variables were available only in plaque patients; healthy controls were therefore not included in this supplementary comparison.
